# Allosteric Modulators of Dopamine D_2_ Receptors for Fine-Tuning of Dopaminergic Neurotransmission in CNS Diseases: Overview, Pharmacology, Structural Aspects and Synthesis

**DOI:** 10.3390/molecules28010178

**Published:** 2022-12-25

**Authors:** Agnieszka A. Kaczor, Tomasz M. Wróbel, Damian Bartuzi

**Affiliations:** 1 Department of Synthesis and Chemical Technology of Pharmaceutical Substances with Computer Modeling Laboratory, Faculty of Pharmacy, Medical University of Lublin, 4A Chodźki St., PL-20093 Lublin, Poland; 2 School of Pharmacy, University of Eastern Finland, Yliopistonranta 1, P.O. Box 1627, FI-70211 Kuopio, Finland; 3 Science for Life Laboratory, Department of Cell and Molecular Biology, Uppsala University, SE-75124 Uppsala, Sweden

**Keywords:** allosteric modulation, dopamine D_2_ receptor, G protein-couple receptors, molecular modeling, drug synthesis

## Abstract

Allosteric modulation of G protein-coupled receptors (GPCRs) is nowadays a hot topic in medicinal chemistry. Allosteric modulators, i.e., compounds which bind in a receptor site topologically distinct from orthosteric sites, exhibit a number of advantages. They are more selective, safer and display a ceiling effect which prevents overdosing. Allosteric modulators of dopamine D_2_ receptor are potential drugs against a number of psychiatric and neurological diseases, such as schizophrenia and Parkinson’s disease. In this review, an insightful summary of current research on D_2_ receptor modulators is presented, ranging from their pharmacology and structural aspects of ligand-receptor interactions to their synthesis.

## 1. Introduction

G protein-coupled receptors (GPCRs) are among the most important drug targets; about 60% of drugs under development and 36% of drugs on the market target GPCRs [1,2]. Among the 19 marketed drugs with the highest sales revenues at their peak year, seven target GPCRs and, in particular, three of them are antipsychotics [3,4]. In 2017 six out of twenty medicines with the highest global sales were GPCR-targeting drugs (clopidogrel, montelukast, valsartan and three antipsychotics: aripiprazole, olanzapine and quetiapinie) [5]. Furthermore, GPCRs are currently the most intensively investigated drug targets in the pharmaceutical industry as they are targeted by about 50% of recently launched drugs [6]. In spite of current narrowing of the pharmaceutical pipeline, the GPCR field remains active, which can be exemplified by FDA approval of two novel antipsychotics only in the year 2015, i.e., brexpiprazole and cariprazine [7] which are dopamine D_2_ receptor partial agonists falling into the same category as aripiprazole. In 2019, FDA approved lumateperone, which is a butyrophenone atypical antipsychotic with a multi-target mode of action [8].

Classically, the action of GPCRs is described by the ternary complex model [9], which implies that a GPCR is involved in a single mechanism (binding with an agonist and signaling via G protein) and only one-dimensional activity is measured [4]. However, GPCR drug action is significantly more nuanced than formerly thought [4], and the lack of control over previously neglected or unrecognized pharmacological factors may very probably result in a lack of efficacy or unfavorable side effects in clinical trials [10]. Moreover, the latest achievements in GPCR pharmacology have challenged the conventional understanding of agonism, antagonism, affinity and efficacy [11]. Novel approaches to tuning the pleotropic action of GPCRs involve allosteric modulators, biased ligands, GPCR heterodimer-targeting compounds, manipulation of polypharmacology, receptor antibodies and tailoring of drug molecules [4,11].

Allosteric ligands interact with binding sites different from orthosteric sites, which are sites for interactions with agonists and competitive antagonists [12]. Allosteric binding sites are evolutionarily conserved to a lesser extent than orthosteric binding sites, which makes it possible to design ligands selective for receptor subtypes [13]. Generally, the allosteric modulators are receptor subtype selective. However, in the case of closely related GPCR subtypes with significant sequence identity, it might happen that a modulator acts on more than one receptor subtype. This is the case with dopamine D_2_ and D_3_ receptors, but it is less likely that modulators act on both D1-like (D_1_ and D_5_) and D2-like (D_2_, D_3_ and D_4_) receptors. Allosteric ligands do not activate the receptors (with the exception of allosteric agonists) but only modulate their response by reinforcement (PAM, positive allosteric modulator) or attenuation (NAM, negative allosteric modulator) of the signal of the orthosteric ligand. Silent allosteric modulators (SAMs, also called silent allosteric ligands) bind to the allosteric site but have no effect on receptor activity. The action of allosteric modulators requires the presence of an endogenous compound (or agonist) which binds to the orthosteric site. Allosteric modulators display a number of advantages over orthosteric ligands. The already mentioned selectivity is one of them. Moreover, the effects of allosteric modulators are subjected to saturation, which prevents the possibility of overdosing (the ceiling effect). It is also important that the effects of modulator action are only visible in the presence of an orthosteric ligand, which results in the modulation of only those neurons in the brain where a neurotransmitter was released. The Allosteric Database (ASD, http://mdl.shsmu.edu.cn/ASD/) [14] lists (as for November 2022) 34,731 allosteric compounds acting through 166 different GPCRs. Most modulators are small organic compounds (33,944), and the rest are polypeptides (420) and ions (7). The practical importance of allosteric drugs was confirmed by approval of the first four allosteric modulators of GPCRs. Cinacalet, introduced to the market in 2004, is a positive allosteric modulator of CaS calcium-sensing receptor, which is used to treat secondary hyperparathyroidism (elevated parathyroid hormone levels) as it blocks the secretion of parathyroid hormone [15]. Maraviroc, approved in 2007, is an allosteric antagonist of chemokine CCR5 receptor and is used to treat AIDS [16]. Plerixafor, which gained FDA approval in 2008, is an allosteric antagonist of CXCR4 receptor, developed for the treatment of AIDS [17]. Ticagrelor, approved by FDA in 2011, is an allosteric antagonist of P2Y12 purinoreceptor for the treatment of stroke and acute coronary syndrome undergoing percutaneous coronary intervention [18]. ASD records 369 allosteric drugs for GPCRs. Apart from the four approved drugs, the database lists 340 compounds in preclinical development, 10 compounds in phase 1, 12 in phase 2 and 3 in phase 3 of clinical studies.

In light of the above, at present one of the hot topics in GPCR-oriented drug discovery is the design of allosteric modulators instead of orthosteric ligands [19,20]. Allosteric modulators that target ion channels (eg. nicotinic ion channels) are well-established drugs; however, it is a relatively new field regarding GPCRs. It is also worth emphasizing that among the 20 allosteric drugs approved by the FDA, only a few have been developed applying an allosteric approach (i.e., GPCR targeting drugs mentioned above and trametinib and cobimetinib targeting kinases) [21]. In contrast, the allosteric mode of action of benzodiazepines on GABA_A_ receptors was demonstrated decades after their development in the 1950s [21].

Allosteric modulators of dopamine D_2_ receptors have untapped clinical potential as more efficient and safer drugs for a number of central nervous system (CNS) disorders, including schizophrenia and Parkinson’s disease (PD). There are also recent reports that modulation of dopaminergic system, in particular through D_3_ receptors, may lead to the discovery of treatments for opioid use disorder [22,23].

In this review we focus on the recent progress in the field of allosteric modulators of dopamine D_2_ receptor as modern agents for precise tuning of dopaminergic neurotransmission for the potential treatment CNS disorders. We present synthesis of the modulators, their pharmacology and structural aspects to illustrate the perspectives of future treatment of schizophrenia, PD and other CNS diseases.

## 2. Overview of Allosteric Modulators of Dopamine D_2_ Receptor

### 2.1. Small Molecules

**Figure 1:**
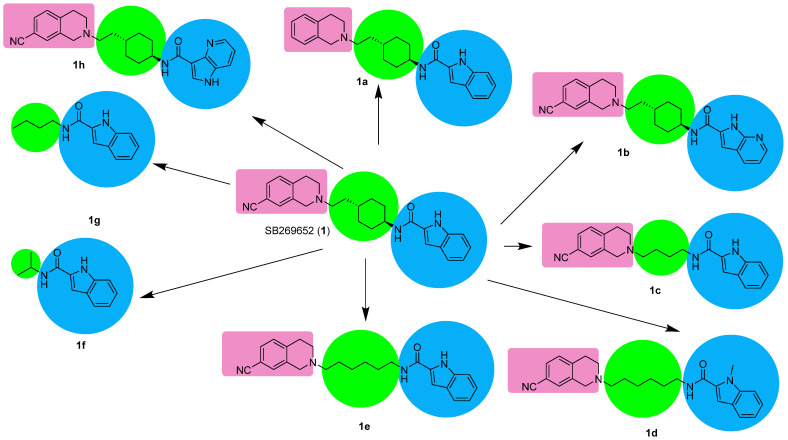
SB-269652 analogues with different motifs. Blue motif binds to the allosteric site, pink binds to the orthosteric site and green is the linker.

One of the most thoroughly investigated small molecule allosteric modulators of dopamine D_2_ receptor is compound **1** (SB-269652), Figure 1, which was first described in 1998 in a patent filled by SmithKline Beecham [24,25]. In 2010 it was reevaluated and reported as D_2_ allosteric modulator [26]. It is the first drug-like allosteric modulator of D_2_ receptor [27]. SB-269652 is a NAM at dopamine D_2_ receptor; however, it contains structural features of an antagonist at this receptor.

Lane et al. [27] investigated the mode of binding of this compound and reported it as a new mechanism of allostery at GPCR dimer. Indeed, by applying a functional complementation system to control the identity of individual protomers within a dopamine D_2_ receptor dimer, they proved that when binding to a dimer is impaired, the mode of action changes from allosteric to competitive. Thus, they concluded that binding of SB-269652 to D_2_ receptor requires the dimer of this receptor. In addition, they demonstrated a ‘bitopic’ pose for SB269652 extending from the orthosteric site into a secondary pocket at the extracellular end of the transmembrane domain, involving TM2 and TM7 [27]. It was found that binding to the secondary binding pocket is required for the allosteric pharmacology of the compound. The new mechanism of allostery at D_2_ receptor relies on the binding of the bitopic ligand to one protomer to allosterically modulate the binding of the orthosteric ligand in the other protomer [27]. It was also suggested that at higher concentration of SB269652 can bind to two allosteric sites within one protomer, which is, however, unfavorable considering its bitopic character [28]. Moreover, it was determined that the sodium ion present within the conserved Na^+^-binding site is necessary for the activity of SB269652 [29]. Draper-Joyce et al. [29] applied fragments of SB269652 and its new derivatives to demonstrate that sodium ions are crucial for the high-affinity interaction of the tetrahydroisoquinoline part of the molecule with the orthosteric binding site. Furthermore, the binding of the indole-2-carboxamide group to the secondary binding pocket is responsible for the range of Na^+^-sensitivity. These findings demonstrate that NAMs of D_2_ receptor may modulate the action of orthosteric ligands synergistically with sodium ions, which opens new avenues for fine-tuning of the receptor activity by novel allosteric drugs. Ågren and Sahlholm suggested [30] that SB269652 induces or selects a D_2_ receptor conformation with higher affinity for this ligand upon binding. Furthermore, the mechanism they observed is dependent on the order of application of dopamine and SB269652, so it is probably competitive in nature or disfavored by simultaneous dopamine binding. Mutagenesis experiments suggest that both the secondary binding site and the orthosteric binding site of D_2_ receptor take part in this mechanism, as the effect of pre-incubation to elevate SB269652 potency was significantly reduced by both E95A and S193A mutation.

In order to design novel efficient antipsychotics, SB269652 can be modified using two main approaches: (i) by preserving its bitopic mode of action and ameliorating its affinity and allosteric effect across dimers at D_2_ receptors, or (ii) by the development of more potent allosteric modulators derived from its indole-2-carboxamide moiety (Figure 1) [28].

Optimization of SB269562 (see Figure 1 and Table 1) was performed by Shonberg et al. [31], Mistry et al. [32] and Kopinathan et al. [33]. Shonberg et al. [31] reported that tetrahydroisoquinoline head group is important to maintain allosteric mode of action and negative cooperativity, in particular when it is C7-substituted with small substituents (halide or cyano group, compound **1b**). Replacement of this moiety with other “privileged structures” for the dopamine D_2_ receptor leads to orthosteric antagonists. Moreover, replacement of cyclohexane linker (compounds **1a** and **1b**) with polymethylene linker allows us to conclude about the dependency of linker length on the allosteric properties of the compounds (flexibility in the spacer region is beneficial for enhancements to functional affinity relative to the rigid cyclohexylene space; an odd number of carbon atoms is unfavorable).

Shonberg et al. [31] also found that indolic hydrogen is crucial for allosteric pharmacology. When the indole core is replaced with the azaindole (compound **1b**) affinity is significantly increased and the negative cooperativity is maintained. Mistry et al. [32] reported compound **1c** with a bitopic mode of action. Moreover, they designed and synthesized a fragment library to analyze SAR and to identify compound **1g,** which exhibited increased negative cooperativity and affinity for dopamine D_2_ receptor. Finally, Kopinathan et al. [33] reported that subtle modifications to the indole-2-carboxamide motif led to a significant increase in functional affinity and cooperativity and a novel action to modulate dopamine efficacy (compound **1h**).

**Figure 2:**
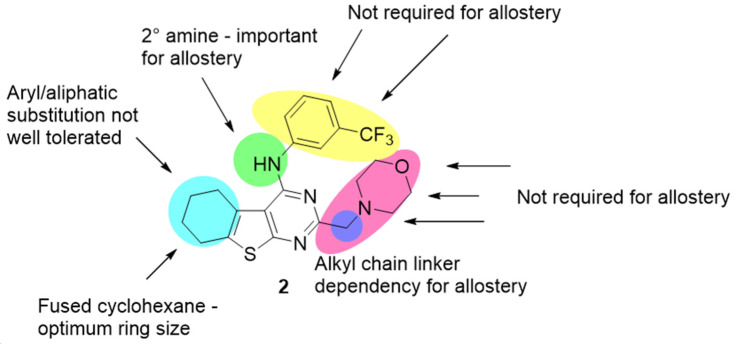
Allosteric D_2_ modulator **2** and its structure-activity relationship. Elaborated based on reference [34].

**Figure 3:**
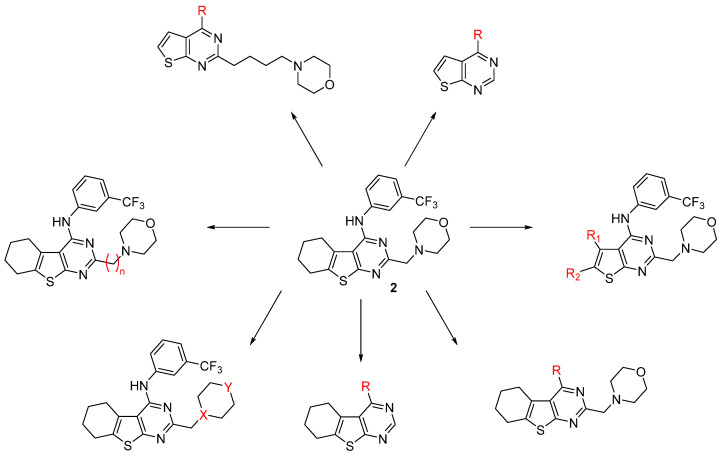
Derivatives of allosteric modulator **2**—directions of modifications.

**Figure 4:**
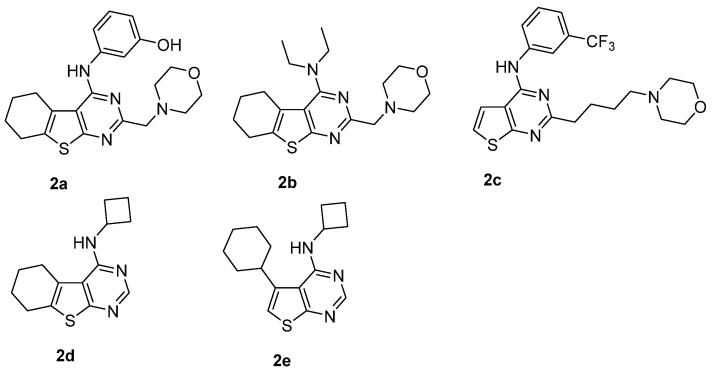
Derivatives of allosteric modulator **2**.

Another D_2_ allosteric modulator **2** (Figure 2) was found in a structure-based virtual screening campaign [34]. This modulator contains a thieno[2,3-d]pyrimidine moiety which has not been previously found in dopaminergic ligands [34]. This compound is a NAM of dopamine efficacy. Fyfe et al. [34] performed an optimization campaign of **2** and found that its main structural groups are responsible for its affinity and cooperativity (see Figure 2 and Figure 3). It turned out that a secondary amine group is important for allostery, as well as that there is an alkyl chain linker dependency for allostery. Moreover, it was found that substitution at the thienopyrimidine at the 5- and 6-positions leads to derivatives with divergent cooperativity profiles. The obtained derivatives displayed a 10-fold improvement in functional affinity, as well as increased negative cooperativity with dopamine affinity and efficacy; see Figure 4 and Table 2 [34].

In the subsequent work, the same research group showed that subtle modifications to a thieno[2,3-d]pyrimidine scaffold yield negative allosteric modulators of D_2_ receptor [35]. They obtained NAMs with divergent affinity/cooperativity profiles (see Figure 4 and Table 2). First, they evaluated the effect of the type of amine substituent at the 4-position in the 5,6,7,8-tetrahydrobenzo[4,5]thieno[2,3-d]pyrimidine scaffold. Next, they studied various substituents at the 5/6-fused cyclohexane moiety, together with the most favorable amine types at the 4-position.

**Figure 5:**
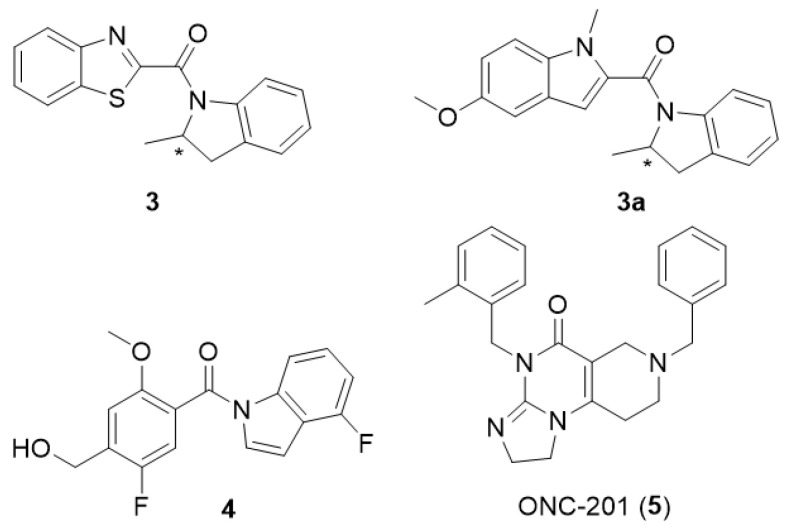
Allosteric modulator **3** and its derivative **3a** suggested as a silent allosteric modulator (SAM). Allosteric modulators **4** and **5**. Chiral carbon is indicated with an asterisk.

As a result of another HTS campaign carried out at UCB Biopharma, a positive allosteric modulator of D_2_ receptor **3**, see Figure 5 was identified [36]. It was a benzothiazole derivative and in its racemic form, it behaves as a PAM of D_2_ receptor. The R enantiomer potentiates the effects of dopamine while the S enantiomer attenuates the effects of the PAM and the effects of dopamine. The effect of enantiomers of compound **3** on the saturation binding of [^3^H]-dopamine to the human dopamine D_2_ receptor is shown in Table 3. Żuk et al. [37] performed molecular dynamics simulations, which confirmed that the R enantiomer of compound **3** is a PAM of the D_2L_ receptor, while its S enantiomer is a NAM. Moreover, the authors obtained a derivative of compound **3**, compound **3a** (see Figure 5) as a racemic mixture and based on the principal component analysis (PCA), they hypothesized that both enantiomers of compound **3a** behave as silent allosteric modulators, which was in accordance with in vitro studies; however the allosteric parameters for this compound are not available.

Scientists at UCB went on and identified another compound **4** as a more potent and efficacious PAM (see Figure 5) [36]. This PAM potentiated the effect of dopamine by an E_max_ of 89% over basal, which is more than the R enantiomer of compound **3** (57%). The effect of enantiomers of compound **4** on the saturation binding of [^3^H]-dopamine to the human dopamine D_2_ receptor is shown in Table 3. This PAM was suitable for behavioral studies, so the authors also investigated its in vivo effects on L-dopa turning behavior. The compound (at the dose of 30 mg/kg i.p.) statistically increased the L-dopa-induced contralateral rotations in the unilateral 6OHDA lesion model in rats. It indicates the possibility of application of compound **4** for the treatment of Parkinson’s disease.

Recently, another D_2_ allosteric modulator has been discovered. ONC-201 (**5**), Figure 5, is a first-in-class imipridone-based derivative currently in clinical studies for the treatment of gliomas and other cancers [38]. Functional parameters for ONC201 antagonism of D_2_R-mediated signaling are shown in Table 4. ONC201 antagonizes the D_2_ receptor with novel bitopic and negative allosteric mechanisms of action, which makes it possible to apply it in the treatment of psychiatric diseases, such as schizophrenia.

### 2.2. Peptidomimetics

**Figure 6:**
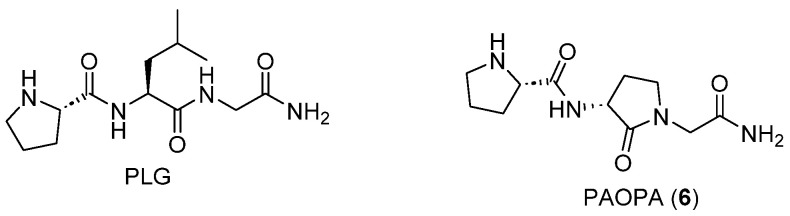
Chemical structures of PLG and PAOPA.

L-Prolyl-L-leucylglycinamide (PLG, Figure 6) is a CNS neuropeptide which has the ability to modulate D_2_ receptors [39]. It is currently termed melanostatin (MIF-1) [40]. It potentiates the binding of agonists to this receptor (E_max_ of 93.6 ± 4.4% for dopamine) [41,42,43,44] with no effect on antagonist binding; see Table 5 [41]. PLG served as a template for development of (S)-N-((R)-1-(2-amino-2-oxoethyl)-2-oxopyrrolidin-3-yl)pyrrolidine-2-carboxamide **6** (PAOPA), its conformationally constrained analog (Figure 6). PAOPA is also a PAM at D_2_ receptor which has the ability to increase dopamine binding; see Table 5 [45]. In behavioral studies, PAOPA (at the dose of 1 mg/kg) prevents NMDA receptor antagonist (MK-801)-induced deficits in social interaction in the rat model [46] and prevents and reverses behavioral and biochemical abnormalities in an amphetamine-sensitized animal model of schizophrenia [47]. It was also shown that PAOPA (at the dose of 1 mg/kg) has a potential for schizophrenia treatment demonstrated in phencyclidine model of schizophrenia in rats [48].

**Figure 7:**
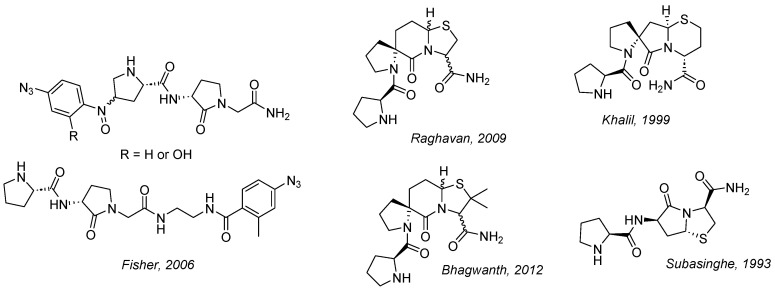
Examples of PLG-derived peptidomimetics.

Multiple other analogues of PLG were designed and synthesized. Notable examples include incorporating 4-azidobenzoyl and 4-azido-2-hydroxybenzoyl photoaffinity-labelling moieties [49], constraining conformation into spiro bicyclic scaffold [50,51], bicyclic thiazolidine scaffold [52], changing the size of the thiazolidine and lactam in constrained ring systems (Figure 7) [53]. Among these compounds D_2_ receptor PAMs and NAMs have been identified. An excellent review outlining chemistry of known peptidomimetics up to 2013 has been published by Bhagwanth et al. [54].

**Figure 8:**
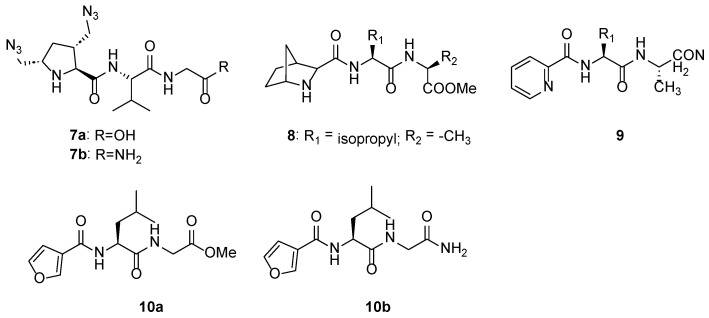
Further examples of PLG-derived peptidomimetics.

Further studies involved generating lower homologs by one carbon atom, replacing leucine with valine [55]. Here compounds **7a** and **7b** (Figure 8) were obtained. These compounds potentiated the binding of the dopamine receptor agonist [^3^H] NPA to dopamine D_2S_ receptor at concentrations between 10^−12^ and 10^−9^ M, similarly to the results for PLG in this assay, which allows us to conclude that they are PAMs of D_2S_ receptor. More recent efforts were related to increasing hydrophobicity of PLG [56]. However, no pharmacological data are available for these compounds. Another attempt at constructing constrained analogues was based on introducing 2-azanorbornane scaffold [57]. A statistically significant increase (*p* < 0.05) in the [^3^H]-NPA response was found for compound **8** (see Figure 8) at 10 pM and 100 pM which indicates that this compound is PAM at D_2_ receptor. The maximum effect was 15 ± 6% at 10 pM. A different strategy by the same group relied on incorporation of picolinoyl group as heteroaromatic proline surrogate [44]. Compound **9** (see Figure 8) obtained within this series produced a statistically significant increase in the maximal [_3_H]-NPA response at 0.01 nM (11.9 ± 3.7%) which indicates it is a PAM at D_2_ receptor. Recently this group elaborated new PAMs of D_2_ receptor based on the bioisosteric replacement of proline to 3-furoic acid in PLG (MIF-1) [40]. They obtained two potent MIF-1 analogues, methyl 3-furoyl-L-leucylglycinate (**10a**) and 3-furoyl-L-leucylglycinamide (**10b**), (Figure 8). PAMs **10a** and **10b** had up to 2-fold and 4.3-fold increase in the EC_50_ of dopamine, respectively. The EC_50_ of **7a** is 0.28 μM and that of **7b** is 0.13 μM. E_max_ of both compounds is 100%. These compounds share the same central dipetide as MIF-1 and changing it results in the loss of PAM activity. Furthermore, the C-terminal carboxamide in **10a** is superior to the methyl ester functional group (**10b**), but is not required for the PAM activity. The lack of a C-terminal carboxamide in **10a** refutes the hypothesis that type II β-turn conformation is necessary for bioactivity as suggested by molecular modeling data.

## 3. Computational Methods to Study Allostery in GPCRs—Dopamine Receptors as an Example

Allosteric modulation is an elusive phenomenon, details of which are difficult to elucidate with in vitro experiments. While there are several X-ray structures of some GPCRs revealing the locations of allosteric sites, especially in chemokine receptors [12,58], clues for allosteric pockets in most other superfamily members, at least so far, could only be derived from indirect experimental evidence or computational methods [59]. While development of the Cryo-EM technique has boosted progress in the field, still less than 10% of available experimentally solved GPCR structures contain allosteric ligands [13]. Experimental explanation of mechanisms underlying allostery is also challenging. For instance, it is suggested that an entropic component may play important role in allosteric signal transmission, allowing modulation without changes to the protein’s shape [60,61,62]. While experimental methods like nuclear magnetic resonance (NMR), double electron-electron resonance (DEER) or Förster resonance energy transfer (FRET) can help to understand protein dynamics upon modulation to some extent [63], in silico tools are the method of choice to gain atomic-level insight into mechanisms of protein function, including pathways of allosteric signal transmission [64].

Computational methods are useful in locating the allosteric site, as well as in elucidation of allosteric mechanisms and signal transmission, which can be further used for the design of either novel small-molecule modulators, or modified proteins with particular signaling properties [65]. As in silico methods commonly used for investigation of allostery in proteins were extensively described in a number of papers published recently, this section provides only short description of available tools and approaches, with particular emphasis on the methods used to study allostery in dopamine receptors and closely related rhodopsin-like GPCRs. For more detailed information on available methods, the reader is referred to appropriate reviews [66,67,68,69,70,71,72].

One possible area of study on allostery, where computers can be successfully employed, is an allosteric pocket search. Identification of possible allosteric pockets suitable for targeting by small organic molecules is crucial for the design of novel modulators, as well as for understanding the structure-activity relationships of those already known, which provides a solid basis for design of improved derivatives.

As described in an excellent review [72], some hints can be deduced from the protein sequence alone. Assuming that allosteric sites and pathways are evolutionarily conserved, their location can be calculated from multi-sequence alignments (MSA) by coevolution analysis. Such an approach was used to identify allosteric communication in D_2_ dopamine receptor [73]. In this study, Sung et al. identified a number of residues participating in the allosteric signal transmission within the receptor, a role of which was subsequently tested in vitro. The sequence-based approaches have important benefits, e.g., modest computational costs. However, there are also apparent disadvantages, including inability to distinguish between residues crucial for transferring allosteric signals from those conserved for other reasons, e.g., structural role. Allosteric pockets can also be found with simple geometry-based algorithms [72], but they do not guarantee that binding to the cavity can induce a signal that could be propagated through the protein of interest.

Improved analysis is possible by including information inferred from protein dynamics. While the main requirement for considering a compound as allosteric is binding away from orthosteric site, the most interesting cases are the molecules that are capable of modifying the protein function upon binding. Consequently, the most interesting binding sites are those from which an allosteric signal can be propagated to regions involved in protein function, e.g., in case of GPCRs, ligand binding sites and/or intracellular effector binding sites. Such sites can be identified by analyzing patterns of protein vibrations, i.e., its normal modes. Normal Mode Analysis (NMA) is a method implemented in several tools, including PARS [74], SPACER [75] or AlloPred [76]. This method allows identification of sites with the greatest potential of affecting dynamics of a protein, at a modest computational cost, even in the absence of any information about the structure of possible modulators [70]. NMA was recently used to investigate vibrations in inactive and active D_2_ receptor models, revealing an important role of TM5 in signal transduction [77].

Protein dynamics can also be implemented in the form of Monte Carlo (MC) [78] or Molecular Dynamics (MD) simulations and related methods. The former is capable of sampling a broad range of protein conformations efficiently, at a cost of losing the time information and, consequently, causation. The latter simulates evolution of a given system in time, which is more computationally demanding, but provides more complete information. Both methods provide a wealth of data to be analyzed. The actual allosteric signal can be sifted by various methods and tools, e.g., MutInf [79], WISP [80], or with Principal Component Analysis (PCA) [81]. Interestingly, in some cases, timescales possible to reach during all-atom unbiased MD with current hardware and software may allow for the observation of large-scale motions associated with allosteric modulation. For instance, Bruzzese et al. used unbiased MD with a long timestep of 4 fs to simulate adenosine A_2a_ receptor, preparing simulation boxes with two different agonists and two different membranes, composed of DOPC (1,2-Dioleoyl-sn-glycero-3-phosphocholine) or DOPG (1,2-dioleoyl-sn-glycero-3-phosphoglycerol) [82]. In this case, a modulatory effect of membrane lipids on receptor activation was observed. The reason for observing very pronounced allosteric effects in a relatively short simulation time was probably the fact that receptor molecules were immersed in pure membranes composed of lipids suspected of modulation, i.e., modulator was provided in a large excess. Allosteric effect of membrane was also observed in earlier, shorter MD simulations of adenosine A_2a_ receptor [83] or μ opioid receptor [84], where the allosteric influence was uncovered with subsequent PCA. In the former, the effect of different head groups (phosphatidylcholine vs. phosphatidylethanolamine) was investigated, uncovering apparent differences in behavior of loop regions upon immersion in different lipid environments [83]. In the latter, different cholesterol concentrations in POPC (1-palmitoyl-2-oleoyl-sn-glycero-3-phosphocholine) were considered in comparison to a raft-like composition, containing POPC, POPE (1-palmitoyl-2-oleoyl-phosphatidylethanolamine), sphingomyelin and cholesterol, which was eventually chosen to study receptor allosteric modulation by three terpenoid negative modulators: salvinorin A, cannabidiol and tetrahydrocannabinol [84].

Another application of all-atom MD for studies on allostery was the seminal work of Dror et al. [85]. Simulation of binding of several allosteric modulators (gallamine, alcuronium, strychnine, C7/3-phth and dimethyl-W84) to M2 muscarinic receptor was possible due to availability of Anton, a high-performance computing facility designed especially for the purpose of MD simulations [86]. The set of 10 μs simulations allowed us to predict the allosteric binding site and the modulators’ binding mode at the extracellular vestibule of the M_2_ receptor.

While the above-mentioned papers have shown that all-atom unbiased MD simulations can provide reliable data describing allosteric processes, nowadays, supervised or enhanced sampling methods are usually employed to study allostery at a lower computational cost [87,88,89]. As an example, binding of an allosteric modulator BMS-986187 to δ opioid receptor was simulated by Shang et al. with a metadynamics-based method [90]. In metadynamics, additional history-dependent potentials are used in order to improve sampling of higher-energy states and facilitate overcoming energy barriers. In the work of Shang et al. an allosteric site was found to be located at the top of transmembrane helices (TM): TM1, TM2 and TM7. These findings are consistent with other MD-based studies on allosteric modulation of a closely related μ opioid receptor [84,91], where the important role of 7.35 residue was underlined. Residue at the 7.35 position is also a part of allosteric binding pocket at dopamine D_2_ receptor [34,92]. While opioid and dopamine receptors belong to different groups within the rhodopsin-like family according to the GRAFS classification (group γ and α, respectively) [93] and classification by Pelé et al. (Group G1 and G2, respectively) [94], they have some common features. In particular, while insertion/deletion in TM2 puts opioid receptors in a group together with chemokine and somatostatin receptors, only opioid receptors in this group contain the key aspartate 3.32 in the binding pocket, serving as an anchor for protonated amine moieties in the way seen in aminergic receptors. Notably, the existence of common allosteric pocket in opioid receptor subtypes was recently postulated [59]. Therefore, the overlap of allosteric pocket location in opioid and dopamine receptors may reflect common allosteric mechanisms.

Both biased and unbiased MD simulations were used by Selent et al. to investigate the entry of sodium ions into D_2_ dopamine receptor [95]. The study included one microsecond-scale unbiased simulation performed with ACEMD [96], as well as several short unbiased and biased simulations. Analysis of trajectories has shown that sodium ions enter the receptor from the extracellular side, bind at the conserved D2.50 residue and affect the conformation of the conserved W6.48. Notably, the study supported previous reports on putative allosteric sodium binding site at the conserved aspartate [97], before publication of 1.8 Å structure of δ opioid receptor, confirming location of the allosteric sodium at that site [98].

Recently Żuk at al. [37] studied interactions of PAM, NAM and SAM (enantiomers of compounds **3** and **3a**, Figure 5) with previously constructed models of D_2_ receptor in active conformation in complex with respective G proteins [99]. In this work molecular docking and all-atom MD simulations in native-like membranes were followed by PCA analysis. PCA allowed the conclusion that the most significant modulator-induced receptor rearrangements are observed at TM7. TM7 bending at the conserved P7.50 and G7.42 was found. Next, the presence of the positive allosteric modulator affected the W7.40 conformation. In the subsequent studies Żuk et al. [100] investigated the role of lipids for allosteric modulation of D_2_ receptor. They found that there is a significant interplay between the membrane, G proteins, receptor and modulators.

Interesting insights into allostery at dopamine receptors were also provided by various techniques of molecular docking. It is another computational tool that can be useful in the identification of allosteric sites. In the absence of any prior information on possible regions of interaction, blind docking can be used [101,102,103]. If there are any experimental data to guide the process, regular flexible docking to the suspected area can be used [67]. Allosteric sites can be located in the flexible regions of a protein, which can be a challenging task for docking algorithms. However, if an allosteric site is located in proximity to the orthosteric one, docking of a bitopic ligand can reveal its location. The first known allosteric modulator of D_2_ and D_3_ receptors, SB269652 [26] is an example of such a situation. With molecular docking followed by molecular dynamics simulations, supported by various experimental assays, Lane et al. have shown that the modulator binds to the orthosteric binding site with its tetrahydroisiquinoline (THIQ) moiety, while its indole group preferentially binds to the secondary pocket (SP) [27]. The study used a homology model of D_2_ receptor built on a D_3_ receptor template. Further, an Induced-Fit Docking (IFD) [104] approach was used to find possible binding poses, which were further assessed with molecular mechanics/generalized Born surface area (MM-GBSA) approach [105]. Docking poses were refined via MD performed with Desmond [106]. Their findings were consistent with previous studies on selectivity determinants of a compound R22 and its derivatives at D_2_ and D_3_ receptor models [107], applying IFD with subsequent MD simulations. These conclusions were surprising, as SB269652 was earlier found to act purely as an allosteric compound [26], while molecular modelling and interaction studies indicated occupation of the orthosteric pocket [27]. Further experiments with a functional complementation assay led to the conclusion that allosteric effect is exerted only in the receptor dimeric form, when the modulator bound to one subunit modulates ligand binding to the second subunit [27]. Studies on SB269652 were later pushed forward by the same team, with a similar molecular modelling protocol, supporting in vitro investigation of newly synthesized compounds [108]. The new research revealed the crucial role of interactions with E2.65 residue in activity of the modulator derivatives, regardless of deploying competitive or allosteric mechanisms. The same group presented also a more detailed computational analysis of the modulator binding at D_2_ and D_3_ receptors, additionally using the Markov state model analysis, which allowed the enhancement of sampling [109]. These extended simulations revealed that the hydrogen bond of the modulator with E2.65 is not maintained for most of the simulation time, and E2.65A mutation changes the modulator properties by affecting shape and dynamics of the allosteric binding pocket.

Another application of IFD in dopamine receptors allostery studies was recently reported by Wang and co-workers. In their work, molecular docking was used as a complimentary approach together with functional assays on D_1_ receptor chimeras and mutants, aiming at identification of the DETQ binding site [110]. After narrowing the search area with experimental studies, molecular docking to the region of the second intracellular loop (icl2) of the D_1_ receptor homology model was performed, which allowed identification of interacting residues, i.e., V119, W123, R130 and L143. Mutation of these residues confirmed their role in modulator binding.

One of the important applications of molecular docking is structure-based virtual screening (VS). It is very useful in searching for novel lead compounds. In recent years, some examples of application of VS to dopamine receptors were reported [92,111]. In particular, in a study published in 2013, Lane et al. performed a screening docking to two D_3_ receptor models, i.e., in the presence or absence of dopamine in the orthosteric binding pocket [92]. Before docking, models were improved with ligand-guided receptor optimization procedure (LiBERO) [112]. Screening against the dopamine-bound receptor was expected to provide candidates for novel allosteric modulators. In contrast to SB269652, none of the 25 candidate compounds had a protonable nitrogen atom, and two of them were confirmed as negative allosteric modulators of D_3_ receptor. Moreover, in a recent work, Fyfe et al. studied pharmacology of one of the hits from the study of Lane et al., found by docking to the receptor in absence of dopamine, and provided evidence for its allosteric mode of action [34]. Additionally, the authors performed a series of molecular dockings of the novel modulator and its analogues, using ICM-Pro software [113]. The docking results underlined the importance of the modulator interactions with TM7, in particular with residues 7.35 and 7.36.

Another recent VS study was focused on signaling bias [111]. Männel et al. used Modeller [114] to create a set of homology models of D_2_ receptor on the D_3_ dopamine receptor template. Subsequently, they used DOCK [115] to identify secondary (allosteric) binding pockets most frequently targeted by biased dopamine receptor ligands. Their results were consistent with previous findings about the importance of TM2 and TM7 in allosteric ligand binding, with residues 2.65 and 7.36 frequently interacting with biased bitopic ligands. On the basis of these findings, Männel et al. were able to identify and synthesize 18 compounds, 16 of which were found to be partial agonists of D_2_ receptor. Among hits, one compound with significant bias toward the β-arrestin pathway was found, proving the usefulness of VS in the design of biased GPCR ligands.

Apart from allosteric pocket search, computational methods can provide relevant information about allosteric signal transmission through a receptor molecule. Such data can be very helpful, not only in the identification of spots located along allosteric signaling pathways, and therefore probably sensitive to modulation by small molecules, but also in the design of modulators with desired properties, e.g., affecting appropriate molecular switches [116]. In a very interesting study, Vaidehi and Bhattacharya employed MD simulations of GPCRs followed by mutual information calculations to identify residue pairs with MI values above average [117,118]. Then, by constructing graphs, with residues as nodes and inter-residue contacts forming edges, the allosteric pathways were calculated. Subsequently, pathways were clustered into pipelines on the basis of spatial proximity. Calculations of allosteric pipelines in three different activation states of β_2_ adrenergic receptor revealed different patterns coupled to inactive, intermediate and active receptor structure. In particular, dynamic behavior of the active structure was characterized by the most pronounced pipeline mediated by the TM7 [119]. In a further study, the same methodology was applied to a set of GPCRs, with the aim to reveal common patterns in dopamine D_3_ receptor, histamine H_1_ receptor, M_2_ and M_3_ muscarinic receptors, A_2a_ adenosine receptor, protease-activated receptor 1 and adrenergic β_1_ and β_2_ receptors [118]. Calculations revealed the existence of two common allosteric pipelines: one starting at the second extracellular loop, leading through parts of TM2, TM5 and TM6 to the intracellular region, and the second, leading through TM7 and ending at helix 8. Notably, these findings are in line with other research described above, underlining the important role of TM2 and TM 7 in allosteric signal transmission, and explaining the special role of the secondary binding pocket/allosteric pocket at the extracellular part of TM2 and TM7 in dopamine receptors and related GPCRs.

## 4. Synthesis of Allosteric Modulators of D_2_ Receptor

### 4.1. Small Molecules

**Scheme 1:**
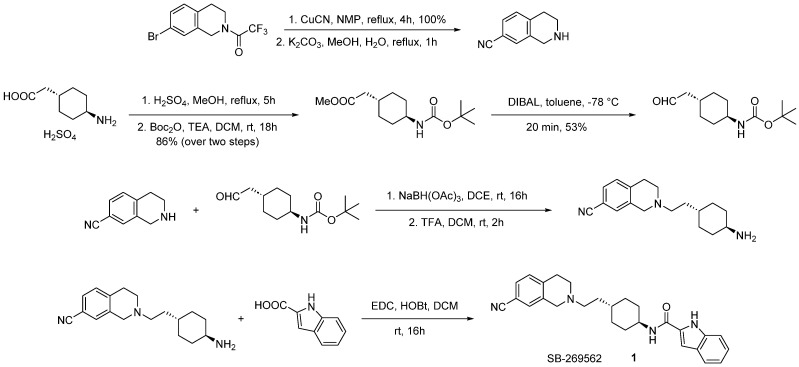
Synthesis of SB-269562.

The synthesis of **1** (SB-269562) was first described in a 1998 patent filled by SmithKline Beecham [24,25]. At the time, this compound was investigated as a ligand for D_3_ receptors. Scientist at SmithKline Beecham envisioned that the target molecule can be obtained by coupling 2-(2-(trans-4-aminocyclohexyl)ethyl)-1,2,3,4-tetrahydroisoquinoline-7-carbonitrile with 1*H*-indole-2-carboxylic acid (Scheme 1). Their synthesis started with preparation of the necessary amine. N-protected bromotetrahydroisoquinoline was converted into a cyano derivative first. Second part of the molecule was elaborated from 2-(trans-4-aminocyclohexyl)acetic acid by esterification, Boc protection and subsequent reduction to obtain tert-butyl (trans-4-(2-oxoethyl)cyclohexyl)carbamate. These two fragments were joined via reductive amination forming the necessary amine required for the final coupling, which was achieved using EDC and HOBt to create an amide bond. Several other protocols have been published since the original procedure involving other coupling reagents such as BOP or HCTU [27,31].

SB-269562 served as an inspiration that spawned numerous analogues different in respect to motifs present in the molecule (Figure 1). Many of the changes introduced involved alkylating nitrogen at carboxamide and indole ring to assess their roles in a hydrogen bond interaction, altering the indole motif to explore the requirement for aromaticity and steric bulk, modifying the spacer group or the tetrahydroisoquinoline core [31]. Truncated analogues were also designed and synthesized, varying at the carboxamide moiety, with the introduction of fluorine to indole at various positions and alterations made to the indole core itself [32]. Other efforts focused exclusively on modifications applied to the indole motif [33]. The synthesis of these compounds utilized previously established chemistry.

**Scheme 2:**
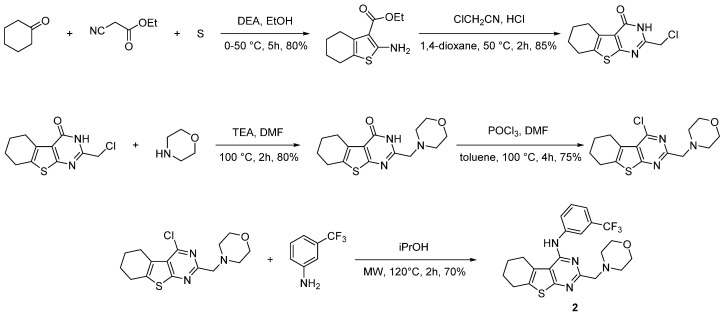
Synthesis of allosteric modulator **2**.

Another D_2_ allosteric modulator **2** was found in HTS campaign. The published synthesis encompasses a five-step linear sequence (Scheme 2) [34]. In the first step, formation of tetrahydrobenzothiophene was realized via Gewald reaction. Subsequent treatment with chloroacetonitrile in an acidic environment afforded the fused pyrimidine ring with the chloromethyl handle. After morpholine alkylation and activation with phosphorus oxychloride, the stage was set for the final nucleophilic aromatic substitution with 3-(trifluoromethyl)aniline to furnish the final compound. The obtained compound bears close resemblance to D_1_ positive allosteric modulator MLS-6585 [120]. Both compounds stem from a common 4,5,6,7-tetrahydrobenzo[b]thiophene scaffold.

Together with the hit compound **2**, the authors synthesized a focused library containing numerous derivatives (Figure 3 and Figure 4). The incorporated changes included exploring the size of the fused carbocyclic ring along with functionalization with single substituents at positions 5 and 6 of the thieno[2,3-d]pyrimidine core. Other modifications relied on removing morpholinomethyl substituent, isosteric replacement of morpholine, varying the length of the methylene linker and transforming the substituent at position 4 in the pyrimidine ring [35,92].

**Scheme 3:**
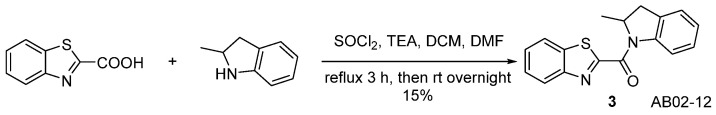
Synthesis of PAM compound **3**.

Compound **3** (AB02-12), a positive allosteric modulator of D_2_ receptor identified in the HTS campaign, is a racemic mixture of benzo[*d*]thiazol-2-yl(2-methylindolin-1-yl)methanone. No synthetic details were provided at the time; however, Bonifazi et al. in their work used the compound as a fragment to construct new D_2_ receptor agonists [121]. Their synthesis of **3** employed simple coupling of activated benzo[*d*]thiazole-2-carboxylic acid with 2-methylindoline (Scheme 3). In an analogous way, Żuk et al. [37] synthesized a derivative **3a** suggested as SAM.

**Scheme 4:**
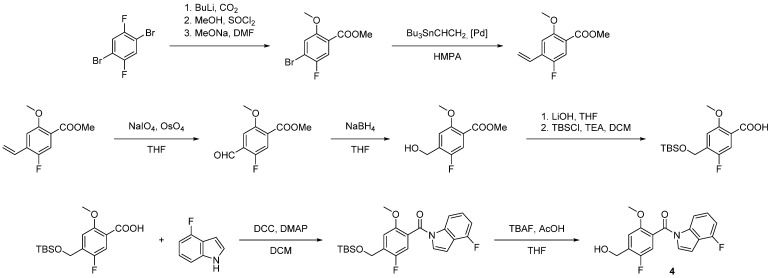
Synthesis of PAM compound **4**.

Scientists at UCB found another compound **4** [36] as a more potent and efficacious PAM. Its synthesis started with 1,4-dibromo-2,5-difluorobenzene, which was carbonylated, esterified and treated with sodium methoxide. The product was subjected to Stille coupling catalyzed by dichlorobis(triphenylphosphine)palladium and the resulting alkene was converted into aldehyde. Reduction with sodium borohydride produced ester alcohol which was first hydrolyzed and then protected. The stage was set for the coupling with 4-fluoroindole, which afforded the final compound after deprotection of the alcohol group (Scheme 4).

**Scheme 5:**
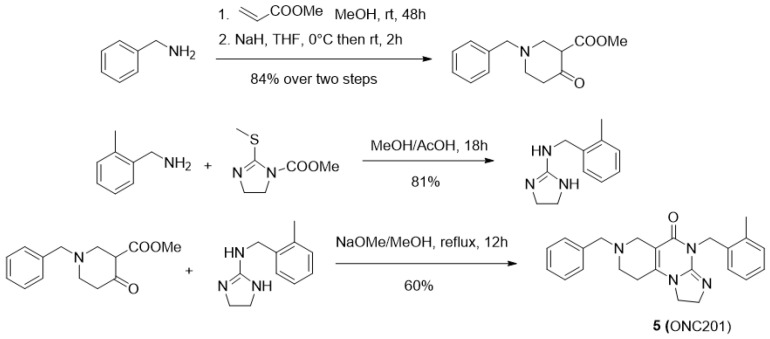
Synthesis of ONC201.

ONC201 was synthesized by coupling the piperidone derivative with the N-substituted aminoimidazoline [122] The former coupling partner was obtained by reacting benzylamine with methyl acrylate via Michael reaction followed by Dieckmann condensation, which afforded the necessary N-benzylpiperidone. The other intermediate was obtained by alkylating 2-methylbenzylamine with S-methyl imidazoline. Both partners, upon condensation, yielded the target compound in satisfactory amounts (Scheme 5).

### 4.2. Peptidomimetics

**Scheme 6:**
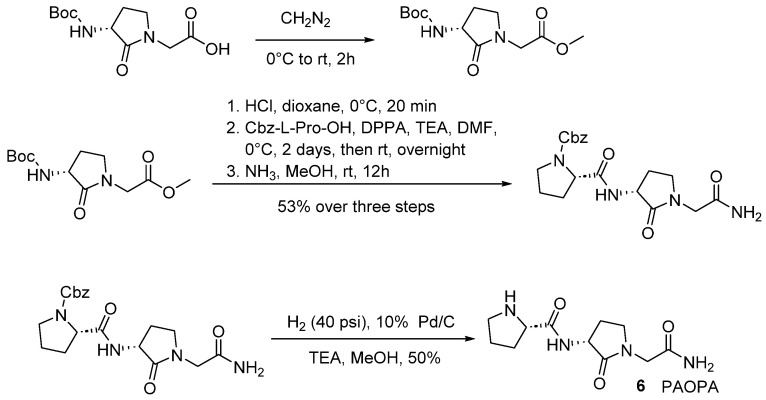
Synthesis of PAOPA.

PAOPA, derived from PLG, was synthesized starting from Boc protected (*R*)-2-(3-amino-2-oxopyrrolidin-1-yl)acetic acid, which was esterified with diazomethane first. Then, in a sequence of reactions, it was deprotected, coupled with Cbz protected L-proline using DPPA and converted into amide. As a final step, hydrogenolysis removed the Cbz group affording the desired compound (Scheme 6) [123].

**Scheme 7:**
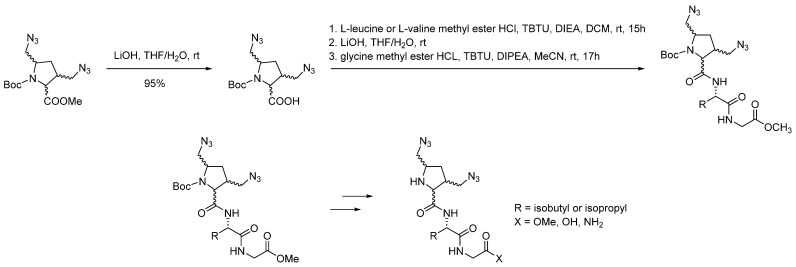
Synthesis of PLG homologs.

Further studies focused on replacing leucine with valine. This modification was also accompanied by connecting two azidomethyl groups to the proline ring. The synthesis of these compounds was initiated by coupling Boc protected 3,5-bis(azidomethyl)pyrrolidine-2-carboxylic acid, which was prepared from the corresponding ester, with either L-leucine or L-valine methyl ester using TBTU as a coupling reagent. Subsequent hydrolysis was followed by the second coupling with glycine methyl ester under analogous conditions. Thus 11 novel compounds were obtained after manipulation of glycine carbonyl group and final deprotection (Scheme 7) [55].

**Scheme 8:**
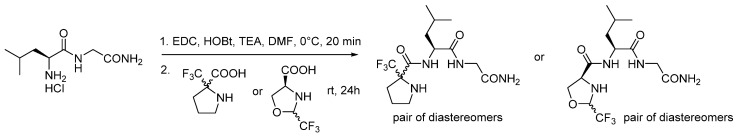
Synthesis of fluorinated analogues of PLG.

In order to increase hydrophobicity of PLG, four novel fluorinated analogous were generated. Using L-Leu-Gly-NH_2_ hydrochloride as a starting material and EDC together with HOBt did not require protected fluorinated amino acid, prevented formation of unwanted diketopiperazines and allowed synthesizing of required compounds as two pairs of diastereomers (Scheme 8) [56].

**Scheme 9:**
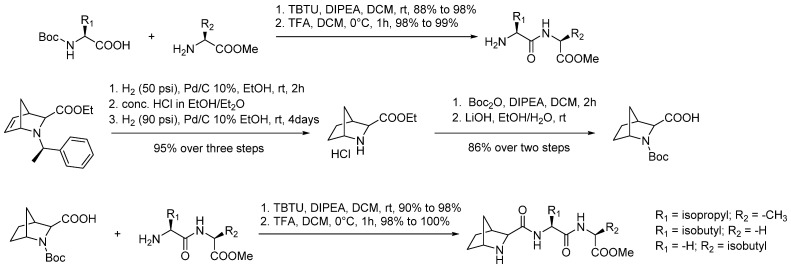
Synthesis of azanorbornane constrained PLG analogues.

Another attempt at constructing constrained analogues was based on introducing 2-azanorbornane scaffold [57]. An aza-Diels-Alder adduct served as a starting material, which was subjected to high-pressure hydrogenolysis affording proline mimetic in enantiopure form. It was then protected with the Boc group and hydrolyzed. The intermediate obtained in that fashion was then subjected to coupling with separately prepared dipeptides. After final removal of the Boc group, three series of analogues were produced, where (a) L-leucine and glycine were replaced by L-valine and L-alanine, (b) the sequence was maintained and (c) the sequence was reversed (Scheme 9).

**Scheme 10:**
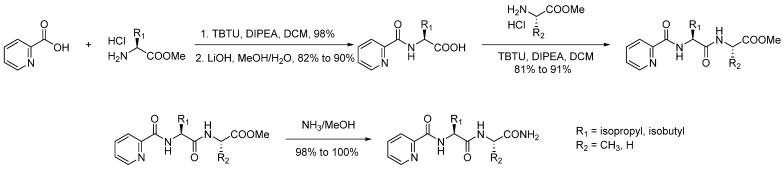
Synthesis of picolinoyl PLG analogues.

A different strategy by the same group relied on incorporation of picolinoyl group as heteroaromatic proline surrogate [44]. The synthesis was based on the previously established chemistry (Scheme 10).

## 5. Conclusions

Allosteric modulators of GPCRs are nowadays one of the hot topics in GPCR-oriented drug discovery as they offer a number of advantages in comparison with classical orthosteric drugs. This is illustrated by the introduction of the first four allosteric modulators to the market. In particular, allosteric modulators of dopamine D_2_ receptor have an untapped potential for the treatment of mental (e.g., schizophrenia) and neurodegenerative (e.g., Parkinson’s disease) disorders. The available modulators belong to two chemical classes: small molecules and peptidomimetics. Although the field is very active, the number of identified modulators is relatively small. It results from the fact that subtle factors govern the activity of allosteric compounds and often a small change in the compound structure results in a complete loss of activity. Here, molecular modeling methods, in particular molecular docking, virtual screening and various techniques of molecular dynamics, may be used to facilitate rational modulator design. However, computer-aided design of GPCRs allosteric modulators is hampered by the existence of multiple allosteric sites visible in available experimental structures of receptor-modulator complexes. Further studies are necessary to decipher the structural requirements of particular binding pockets and design more potent compounds. It should be also stated that despite the progress in GPCR in vitro pharmacology, it is often difficult to detect allosteric properties of novel compounds due to a number of affecting factors, in particular the phenomenon of probe dependence. Future efforts in the field should be focused on the elaboration of molecular modeling tools for allosteric modulators design and improvement of in vitro pharmacology tools to reflect the nuanced picture of GPCRs allosteric modulation. Furthermore, the available synthetic protocols, while capable of producing the desired compounds, can be improved in certain cases where the reported yield is low. Moreover, newer synthetic methods should allow for the generation of analogs for possible SAR exploration. Summing up, although challenging, the design and discovery of D_2_ receptor allosteric modulators is worth an effort, as it may bring a breakthrough in the field of antipsychotics and drugs used to treat Parkinson’s disease.

## Figures and Tables

**Table 1:** Allosteric parameters of SB269562 and its analogues. NA—not available. ^a^ Estimate of the equilibrium dissociation constant determined in a pERK1/2 functional assay; ^b^ Estimate of the net cooperativity factor between the modulator and determined in an pERK1/2 functional assay.

Compound	K_B_^a^, nM	αβ ^b^
SB-269562 (**1**) [27]	776	0.06
**1a** [31]	87	0.091
**1b** [31]	23.4	0.04
**1c** [32]	81	0.05
**1d** [31]	72.4	NA
**1e** [31]	30	0.021
**1f** [32]	4600	0.08
**1g** [32]	6000	0.02
**1h** [33]	0.148	α = 0.048 Β = 0.16

**Table 2:** Examples of derivatives of compound **2**. ^a^ Estimate of the negative logarithm of the equilibrium dissociation constant determined in an cAMP functional assay^. b^ Estimate of the logarithm of the net cooperativity factor between the modulator and dopamine. ^c^ Estimate of the logarithm of the modulatory effect upon efficacy factor induced by the allosteric modulator. Values represent the mean ± SEM from at least three independent experiments performed in duplicate.

Compound	pK_B_^a^ (K_B_) [μM]	Logα ^b^ (α)	Logβ ^c^ (β)
**2** [34]	5.41 ± 0.22 (3.87)	0	−0.55 ± 0.08 (0.28)
**2a** [34]	5.55 ± 0.12 (2.81)	−1.22 ± 0.16 (0.06)	−3.0
**2b** [34]	6.18 ± 0.16 (0.662)	−0.17 ± 0.17 (0.68)	−1.10 ± 0.10 (0.08)
**2c** [34]	5.58 ± 0.10 (1.4)	−0.88 ± 0.16 (0.13)	−1.21 ± 0.16 (0.06)
**2d** [35]	6.25 ± 0.12 (0.57)	−0.72 ± 0.12 (0.19)	−0.65 ± 0.07 (0.22)
**2e** [35]	5.53 ± 0.13 (2.92)	0	−1.55 ± 0.19 (0.03)

**Table 3:** Effects of the stereoisomers of compound **3** and compound **4** on the saturation binding of [^3^H]-dopamine to the human dopamine D_2_ receptor. Data are mean 6 standard deviation from three separate experiments [36].

Parameter	+R Isomer of 3 (10 μM)	+S Isomer of 3 (10 μM)	+Compound 4 (10 μM)
K_d_ [nM]	13 ± 3	17 ± 6	11 ± 4
B_max_ [%control]	137 ± 10	82 ± 16	278 ± 51

**Table 4:** Functional parameters for ONC201 antagonism of D_2_R-mediated signaling. Data are the best fit of grouped data from at least 3 independent experiments, expressed as geometric means [95% confidence interval]. ^a^ The logarithm of dopamine efficacy; ^b^ the logarithm of ONC201 efficacy constrained to −3 to indicate ONC201 displays no detectable agonist efficacy; ^c^ the negative logarithm of dopamine affinity; ^d^ the negative logarithm of ONC201 affinity; ^e^ the logarithm of the binding cooperativity between dopamine and ONC201; ^f^ the logarithm of the magnitude of the allosteric effect of the modulator on the efficacy of the orthosteric agonist, constrained to −3 indicating that ONC201 exhibits a very high level of negative cooperativity; ^g^ the Hill slope [38].

Assay	logτ_A_^a^	logτ_B_^b^	pK_A_^c^	pK_B_^d^	Logα ^e^	Logβ ^f^	Slope ^g^
β-arrestin	1.57 [0.73–2.40]	−3.00	5.26 [4.39–6.14]	5.53 5.42–5.64] (3 μM)	−0.70 [−1.59–0.20] (0.2)	−3.00	1.21 [1.06–1.35]
cAMP	1.68 [1.21–2.23]	−3.00	5.62 [5.05–6.15]	5.64 [5.37–5.92] (2.3 μM)	−0.28 [−0.85–0.34] (0.5)	−3.00	0.88 [0.77–1.02]

**Table 5:** Modulation of [^3^H]spiperone/N-n-Propylnorapomorphine (NPA) at D_2L_ receptors by PLG and PAOPA [45]. K_H_ and K_L_ represent the inhibition constant (K_i_) of agonist, calculated from the high-affinity and low-affinity component of [^3^H]spiroperidol binding, respectively. Percent R_H_ and percent R_L_ are, respectively, the percentage of sites in high- or low-affinity form for the agonist. R_H_/R_L_ is the ratio of the two percentages. Each value is an average of three to four separate experiments ± S.E.M., carried out in duplicate or triplicate. The concentration of PLG used was 1 μM and the concentration of PAOPA was 1 nM. Gpp(NH)p: guanosine 5′-(β,γ-imido)triphosphate.

Condition	Binding Parameter				
	K_H_ [nM]	K_L_ [nM]	R_H_ [%]	R_L_ [%]	R_H_/R_L_
PLG-treated-Gpp(NH)p	0.020 ± 0.001	78.0 ± 5.0	73.0 ± 6.0	27.0 ± 3.0	2.70 ± 0.28
PLG-treated+Gpp(NH)p	0.040 ± 0.002	92.0 ± 8.0	34.0 ± 2.5	66.0 ± 11	0.55 ± 0.040
PAOPA-treated-Gpp(NH)p	0.030 ± 0.002	63.0 ± 4.0	74.0 ± 8.0a	26.0 ± 3.0	2.80 ± 0.19
PAOPA-treated+Gpp(NH)p	0.050 ± 0.003	88.0 ± 9.0	44.0 ± 6.0	56.0 ± 7.0	0.78 ± 0.050

## Data Availability

Not applicable.
